# Poly-Gly Region Regulates the Accessibility of Metal Binding Sites in Snake Venom Peptides

**DOI:** 10.1021/acs.inorgchem.2c02584

**Published:** 2022-08-30

**Authors:** Joanna Wa̧tły, Aleksandra Hecel, Robert Wieczorek, Magdalena Rowińska-Żyrek, Henryk Kozłowski

**Affiliations:** † Faculty of Chemistry, University of Wrocław, F. Joliot-Curie 14, Wrocław 50-383, Poland; ‡ Institute of Health Sciences, University of Opole, 68 Katowicka Street, Opole 45-060, Poland

## Abstract

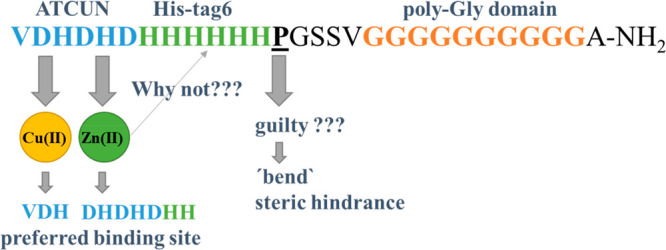

It is supposed that the presence of poly-His regions in close proximity to poly-Gly domains in snake venoms is related to their biological activity; poly-His/poly-Gly (pHpG) peptides inhibit the activity of metalloproteinases during venom storage via the chelation metal ions, necessary for their proper functioning. This work shows that only the histidyl residues from the N-terminal VDHDHDH motif (but not from the poly-His tag) were the primary Zn(II) binding sites and that the poly-Gly domain situated in the proximity of a central proline residue may play a regulatory role in venom gland protection. The proline induces a kink of the peptide, resulting in steric hindrance, which may modulate the accessibility of potential metal binding sites in the poly-His domain and may, in turn, be one of the regulators of Zn(II) accessibility in the venom gland and therefore a modulator of metalloproteinase activity during venom storage.

## Abstract 2

The proline induces a kink of the peptide, resulting in a steric hindrance, which may modulate the accessibility of potential metal binding sites in the poly-His domain and may, in turn, be one of the regulators of Zn(II) accessibility in the venom gland and therefore a modulator of metalloproteinase activity during venom storage.

Snake venoms are a mixture of bioactive peptides, proteins, metal ions, and organic compounds.^1,2^ Snake venom metalloproteinases (SVMPs) account for approximately 30% of all peptides in snake venoms. They are one of the most active mixtures of proteases found in nature, influencing the venom toxicity because of their ability to, e.g., induce hemorrhage, proteolytically degrade fibrin and fibrinogen, or aggregate platelets.^3^ Metalloproteinases are synthesized and stored in the venom gland of snakes in an inactive form.^4^ SVMPs are related to the ADAM (A Disintegrin And Metalloproteinase) family of proteinases that function properly in the presence of metal ions such as (i) zinc, which is essential for their enzymatic activity, and (ii) calcium, which is important for structural stabilization.^5,6^

An extremely fascinating phenomenon of snakes and vipers is total resistance to their own venom. The venom gland is protected because the activity of metalloproteinases in the gland is inhibited by (i) low pH, (ii) chelation of calcium ions with citrate, and (iii) competitive enzymatic inhibition by the tripeptide pyroglutamate–lysine–tryptophan (pEKW). This activity can be restored through dilution or physicochemical changes when the venom is introduced to the victim’s body.^7^

Studies show that also peptides containing poly-His and poly-Gly domains, found in African vipers from the *Atheris* and *Echis* families (Table 1), most likely contribute to inhibition of the proteolytic activity of SVMPs.^7,8^ Poly-His/poly-Gly (pHpG) peptides probably act as metal-ion chelators, trapping/buffering them in the snake venom gland and protecting the host from the destructive effects of SVMPs.^9^ Although our knowledge of the interactions of poly-His peptides with metal ions is rapidly expanding, it still holds many secrets and requires further studies on model systems, especially on those that contain additional interesting motifs in their sequence that potentially bind metal ions [e.g., the amino terminal Cu(II)- and Ni(II)-binding (ATCUN) motif] or indirectly affect the manner of interaction, the structure, or stabilization of the complex (e.g., poly-Gly domain or presence of specific amino acid residues, e.g., proline).

**Table 1:** pHpG Peptide Sequences Identified in *Echis ocellatus* Venom and Related pHpGs from Other Venoms^7^

name	full sequence (abbreviated sequences in parentheses)
pHpG-1 *E. ocellatus*	DHDHDHHHHHHPGSSVGGGGGGGGGG (DHDHDH_(6)_PGSSVG_(10)_)
pHpG-2 *E. ocellatus*	DHDHDHHHHHHPGSSVGGGGGGGGGGA (DHDHDH_(6)_PGSSVG_(10)_A)
pHpG-3 *E. ocellatus*	VDHDHDHHHHHHPGSSVGGGGGGGGGG (VDHDHDH_(6)_PGSSVG_(10)_)
pHpG-4 *E. ocellatus*	VDHDHDHHHHHHPGSSVGGGGGGGGGGA (VDHDHDH_(6)_PGSSVG_(10)_A)
pHpG-1 *A. squamigera* and *Atheris chlorechis*	EDDHHHHHHHHHGVGGGGGGGGGG (EDDH_(9)_GVG_(10)_)
pHpG-1 *Atheris nitschei*	EDDHDHHHHHHHHHHHHGVGGGGGGGGGGA (EDDHDH_(12)_GVG_(10)_A)

In this work, we focus on the VDHDHDHHHHHHPGSSVGGGGGGGGGGA-NH_2_ peptide (pHpG-4), found in the venom of *E. ocellatus* (Table 1), which contains three specific domains: (i) VDHDHD; (ii) six neighboring histidines (His-tag6); (iii) the poly-Gly domain with nine Gly residues at the amidated C terminus.^7^ The first region is a typical ATCUN motif, found in metal-transporting albumins, neuromedins, C and K, histatins, or human sperm protamine P2a.^10^ The motif engages a 4N coordination donor set, including a free NH_2_ terminus, two deprotonated amide N atoms from two amino acids in positions 1 and 2, and an imidazole from a histidine residue in the third position. This specific sequence forms very stable square-planar complexes with Cu(II) and Ni(II) ions composed of fused 5,5,6-membered chelate rings.^11−13^ The second motif present in the sequence is the “His-tag” motif often found in the natural proteins and is also commonly used for protein purification with immobilized metal affinity chromatography (IMAC).^14−17^ This motif has the possibility of binding metal ions in several ways, where a maximum of two His residues may bind the metal (in the sequence with at least three and no more than six His residues).^18−21^ The third motif, consisting of a number of neighboring glycyl residues, does not have any particularly attractive binding donors (apart from the amide N atoms), but it is one of the most flexible polypeptides with the ability to eliminate steric hindrance.^22^ Also, the presence of a proline, which has the ability to “bend” the peptide chain between the poly-His and poly-Gly domains, is noteworthy.^23^

Our previous studies on the pHpG-1 peptide fragments from *E. ocellatus* [Table 1; DHDHDHHHHHHPGSSV-NH_2_ (N-DpH) and Ac-DHDHDHHHHHHPGSSV-NH_2_ (DpH) in Table 2] showed the following: (i) the free amino group plays a significant role in the thermodynamic enhancement of metal-ion binding; (ii) Cu(II) can be coordinated by different sets of imidazole rings before amide N-atom coordination, which takes place at higher pH (around pH 6); (iii) in close proximity to Cu(II) binding sites, a preference for the formation of a 3–10 helical structure exists.^24^ While the pHpG-1 peptide from the venom of *Atheris squamigera* forms extremely thermodynamically stable complexes with Cu(II) ions (even more stable than the albumin-like ATCUN motif) and seems to be correlated *inter alia* with preferences of Cu(II) binding to residues separated by one amino acid and with predisposition to form an α-helical structure formation.^25^ In the case of terminally protected peptides, a significant number of “polymorphic states” were observed in which sets of three different histidyl residues were involved in metal-ion binding, and the metal binding induced the formation of a regular α-helical structure,^9^ similar to the interaction of metal ions with the typical His-tag6 (Table 2), used in IMAC.^18^

**Table 2:** Poly-His and pHpG Peptides Studied Earlier and Compared in This Work

peptide	sequence	ref
His-tag6	Ac-HHHHHH-NH_2_	(18)
pHpG-1 from *A. squamigera*	EDDHHHHHHHHHGVGGGGGGGGGG-NH_2_	(25)
pHG, fragment of pHpG-1 from *A. squamigera*	Ac-EDDHHHHHHHHHG-NH_2_	(9)
N-DpH, fragment of pHG-1 and pHG-2 from *E. ocellatus*	DHDHDHHHHHHPGSSV-NH_2_	(24)
DpH, C- and N-terminal-protected fragment of pHG-1 and pHG-2 from *E. ocellatus*	Ac-DHDHDHHHHHHPGSSV-NH_2_	(24)
pHpG-4 from *E. ocellatus*	VDHDHDHHHHHHPGSSVGGGGGGGGGGA-NH_2_	a

a This work.

Our previously studied peptide, N-DpH (Table 2), turned out to be even more effective in binding Zn(II) ions than pHpG-1 probably because of the suggested participation of the terminal N atom in Zn(II) binding ({3N_im_, NH_2_} donor set).^24^ A comparison of these systems with the Zn(II)-pHG complex (Table 2 and Figure 1) suggests that not only the number and position of the histidyl residues but also the availability of the N terminus and the presence of a poly-Gly chain can have a significant influence on the specificity of the metal–pHpG interactions.

**Figure 1:**
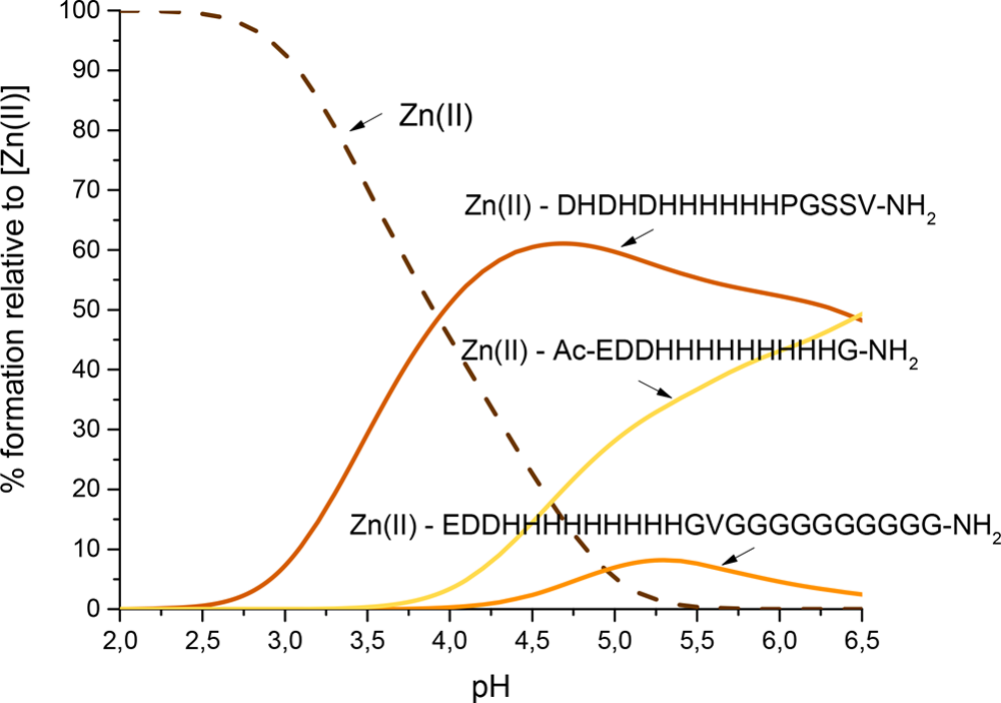
Competition plot for Zn(II) complexes with EDDHHHHHHHHHGVGGGGGGGGGG-NH_2_ (pHpG-1, orange), Ac-EDDHHHHHHHHHG-NH_2_ (pHG, yellow), and DHDHDHHHHHHPGSSV-NH_2_ (N-DpH, brown) peptides. The potentiometric data were taken from refs (9), (24), and (26), respectively.

In this work, we compare the thermodynamic stability and structural properties of Cu(II) and Zn(II) complexes with VDHDHDHHHHHHPGSSVGGGGGGGGGGA-NH_2_ (pHpG-4) to the previously studied peptides containing poly-His and/or poly-Gly motifs. Such knowledge of peptides from snake venoms, which contain poly-His and poly-Gly domains, and their interactions with biologically important metals not only is the key to understanding the role of pHpG peptides in venoms but also provides a solid pillar to the beautiful, basic bioinorganic chemistry of Cu(II) complexes with poly-His and poly-Gly peptide motifs.

Electrospray ionization mass spectrometry (ESI-MS) revealed the formation of equimolar species in the case when 1:1 metal-to-ligand stoichiometry was used (Figures S1 and S2).

Potentiometric titrations allowed one to determine the protonation and stability constants (Figures S3 and S4 and Table S1) and further confirmed the 1:1 stoichiometry. It should be noted that mass spectrometry, like every technique, has its limitations; minor species present in the solution may not always be visible in the spectra because of either the lack of protonation or the fact that, in the conditions necessary to create gas-phase ions, low-affinity species may not always be visible (that is why MS cannot be used as a lone piece of evidence to prove the equimolar stoichiometry). In this case, potentiometry provides a very good support for the equimolar stoichiometry of the detected species, detecting only the formation constants for 1:1 metal-to-ligand species.

The far-UV circular dichroism (CD) spectra of the free ligand show no clear tendency of the ligand to adapt an ordered secondary structure (Figure S5A) but may suggest a small share of the helical structure, which is indicated by a slightly negative signal around 230 nm.^27,28^

The far-UV CD spectrum of the Cu(II) complex at pH 5.5 (Figure S5C) differs from the spectrum of the ligand obtained at the same pH (the signal of the negative peak near 226 nm is slightly increased, and the amplitude of the negative peak near 200 nm is slightly decreased and shifted toward higher wavelengths), which may suggest a higher content of the helical structure (although still small) compared to the free ligand.^27^ A similar effect is observed for Zn(II) complexes (Figure S6).

Cu(II) binds to the studied ligand with a {1N_im_, 2N_am_, 1NH_2_} donor set, characteristic for systems with Cu(II) [or Ni(II)], and peptides with a free N terminus and histidine in the third position above pH 6.9, as confirmed by UV–vis, CD, and electron paramagnetic resonance (EPR) spectroscopy (see Tables S1 and S2 and Figures S4, S5, S7, and S8 and the discussion of the results in the Supporting Information).

In the case of the Zn(II) complex, at physiological pH, the {4N_im_}-coordinated species may be in equilibrium with other complexes, in which two different His residues coordinate the Zn(II) ion (see Table S3 and Figures S6 and S9 and the discussion of the results in the SI).

Density functional theory (DFT) calculations perfectly complement the experimental work and allow one to accurately determine the metal–peptide interactions (Figures S10 and S11 and Tables S4 and S5).^9,18,24^ In the case of the Cu(II)-pHpG-4 system, CuH_7_L and CuH_6_L complexes are 2N type and use H3 and H5 imidazole rings to bind Cu(II) (Figure 2). Interestingly, short six-residue (19–24) unusual polyglycine helical fragments can be detected in CuH_6_L and CuH_7_L complexes (marked in red in Figure 2), similar to those reported previously.^29^ The first 3N-type complex is CuH_5_L; two imidazole N atoms from H3 and H5 and the H3 amide take part in the coordination. CuH_4_L, CuH_3_L, and CuH_2_L are 3N-type complexes that share the same 3N binding pattern, in which D2 and H3 amide N atoms and the H3 imidazole N atom are involved. CuHL is a typical albumine-like complex. The Cu(II) binding to amides of D2 and H3 and to a H3 imidazole N atom is supported by the binding of the V1 amino N atom.

**Figure 2:**
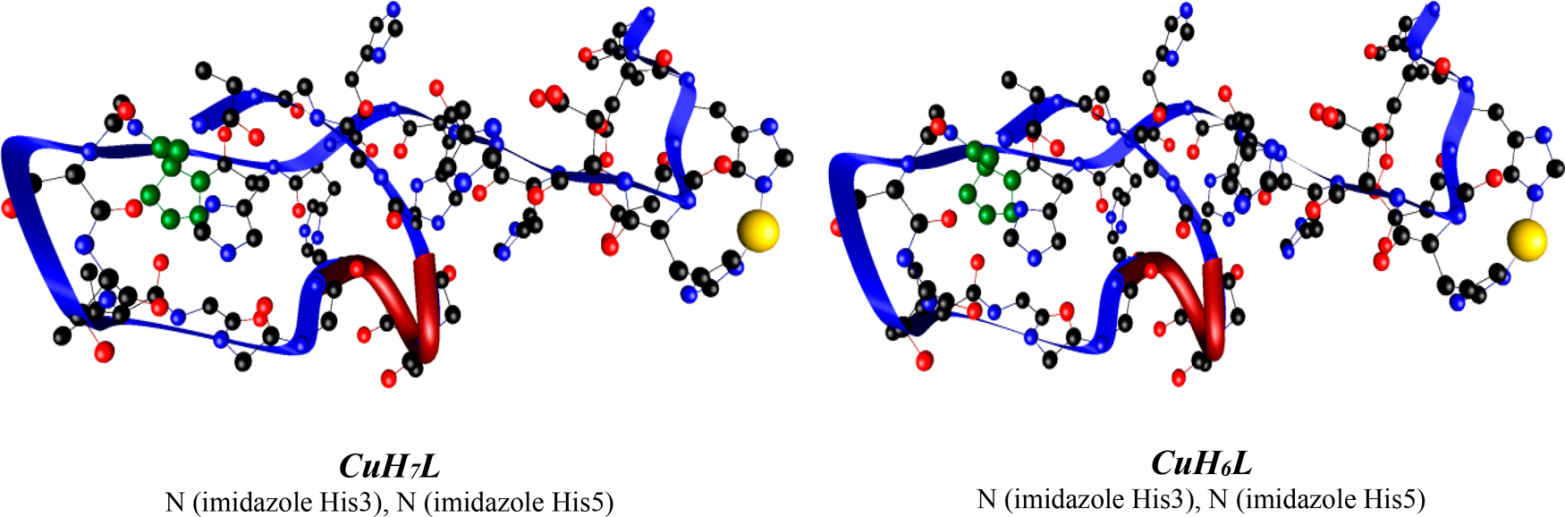
Structures of Cu(II)-VDHDHDHHHHHHPGSSVGGGGGGGGGGA-NH_2_ complexes with 2N_im_ coordination modes. Blue ribbons follow the backbone, and proline is marked in green and Cu(II) ion in yellow.

In the ZnH_6_L (2N) complex, H3 and H5 take part in binding [as in the case of Cu(II)]; the complex is additionally stabilized by two supporting interactions with carbonyl O atoms of H3 and D4. ZnH_5_L is the only complex in which three imidazole rings (of H3, H5, and H7) are involved in Zn(II) binding; the N-type set of interactions is supported by one Zn–O bond, where the O atom comes from the carbonyl group of H3. Interestingly, the ZnH_4_L species can exist in two forms (A and B), where imidazole rings of H5 and H7 for ZnH_4_L (A) and H3 and H7 for ZnH_4_L (B) are involved (Figure S11 and Table S5). All metal–ligand interactions are discussed in the *DFT Calculations Complete the Experimental Results* section in the SI.

A comparison of the thermodynamic stabilities of snake-venom-derived peptide complexes (as discussed in the SI and shown in Figures S12–S14 and Tables S6–S8) shows the importance of polymorphic states and the formation of a helical structure in the presence of metal ions. The presence of (i) proline, (ii) poly-Gly, and (iii) an ATCUN motif and the number of His residues in the pHpG-4 sequence strongly stabilize the Cu(II) complexes above pH 7. A different trend in the affinity of metal-ion coordination is observed in the case of Zn(II) complexes; the presence of the proline residue and poly-Gly sequence in the peptide significantly reduces the thermodynamic stability of Zn(II) complexes.

In conclusion, various analogues of the peptide from the snake venom *E. ocellatus* show different coordination chemistry, depending on the available coordination sites and the arrangement of amino acid residues in the sequence; especially the arrangement of His residues (in the second or third position) in the case of N-terminal free peptides significantly affects the thermodynamic and structural properties of the complexes formed with Cu(II) and Zn(II) ions.

Four different types of coordination modes of Cu(II) by the studied ligand depending on the pH value were suggested. pHpG-4 with an ATCUN motif has a unique affinity for Cu(II) ions. The imidazole N atom of H3 is the main anchoring site for the metal ion, and as the pH increasess, Cu(II) is bound to one imidazole N atom, two amide N atoms, and the N atom from the amino group. The deprotonation of two amide groups and the binding of the metal ion occurs in a very narrow pH range (5.5–6.5). The albumin-like complex with a {1N_im_, 2N_amide_, NH_2_} binding mode is very stable and dominates above pH 6.90. It is worth noting that the pHpG-4 peptide, containing both ATCUN and His-tag motifs (VDHDHDHHHHHHPGSSVGGGGGGGGGGA-NH_2_), forms a 4N complex at higher pH, like in the case of the peptide without the poly-His chain (2 or even almost 3 units of pH higher).

Studies of Zn(II) complexes with the pHpG-1 peptide from *A. squamigera* on its fragment, the pHG peptide, and also the N-DpH peptide (fragments of pHG-1 and pHG-2 from *E. ocellatus*) show that, despite the similar number of His residues (nine, nine, and eight, respectively), their relative positions and the presence of the poly-Gly domain are crucial for the affinity of Zn(II) binding.

The results suggest that it is not the high stability of the complexes but, on the contrary, their low or moderate stability that may be crucial for the role in inactivating the venom in the venom gland. The low stability constants of the Zn(II)-pHpG-4 complex, despite the presence of a number of His residues in sequence, are, most likely, due to the presence of proline, followed by the poly-Gly domain, which most probably forms a kind of steric hindrance (a “curtain”), covering the imidazole residues from the His-tag domain, which would otherwise be highly willing to bind Zn(II) ions. In this way, only the histidyl residues from the VDHDHDH motif are able to bind Zn(II). Experimental and theoretical studies suggest the presence of a few Zn(II) complexes in equilibrium (coordinated via two or a maximum of three His residues from this region).

Because of the presence of the proline residue between the poly-His and poly-Gly motifs, the peptide is able to “bend”, and, presumably, this proline-induced structural kink of the peptide results in steric hindrance, which regulates the accessibility of potential metal binding sites in the poly-His domain, which may, in turn, be one of the regulators of Zn(II) accessibility and therefore a modulator of metalloproteinase activity during venom storage in the venom gland.

## Supporting Information Available

Experimental section, results and discussion (for Cu(II)-pHpG-4 complexes, ESI-MS, potentiometric titrations, UV–vis, CD and EPR spectroscopy, and DFT calculations, and for Zn(II)-pHpG-4 complexes, ESI-MS, potentiometric titrations, far-UV CD spectroscopy, and DFT calculations), a comparison of the complex thermodynamic stabilities, and Figures S1–S14 and Tables S1–S8 (PDF)
