# Supplementary material for: Poly-Gly Region Regulates the Accessibility of Metal Binding Sites in Snake Venom Peptides
Source: Inorg Chem. 2022 Aug 30;61(36):14247–51. doi: 10.1021/acs.inorgchem.2c02584 (PMC9472272; doi:10.1021/acs.inorgchem.2c02584)
Supplement: Supplementary file 1 — ic2c02584_si_001.pdf [file ic2c02584_si_001.pdf]

Supplementary Information

Poly-Gly region regulates the accessibility of metal binding sites in snake venom peptides

Joanna Wątły,[1] Aleksandra Hecel,[1] Robert Wieczorek,[1] Magdalena Rowińska-Żyrek,[1] Henryk Kozłowski[1,2]*

[1] Faculty of Chemistry, University of Wroclaw, F. Joliot-Curie 14, 50-383 Wroclaw, Poland

[2] Institute of Health Sciences, University of Opole, 68 Katowicka St., 45-060 Opole, Poland

*e-mail: joanna.watly@chem.uni.wroc.pl

EXPERIMENTAL SECTION

Materials

VDHDHDHHHHHHPGSSVGGGGGGGGGGA-NH_2 (pHpG-4) peptide was purchased from KareBay Biochem (USA) with certified purity of 98%. Cu(II) and Zn(II) perchlorates were extra-pure products (Sigma-Aldrich). The concentrations of stock solutions of these salts were determined by inductively coupled plasma mass spectrometry. The carbonate-free stock solutions of 0.1 M NaOH were purchased from Sigma-Aldrich and then potentiometrically standardized with the potassium phthalate (99.9% purity) as a primary standard. The $HClO_4$ stock solution was prepared by diluting concentrated $HClO_4$ (Sigma-Aldrich) and then standardized with NaOH. All of the sample solutions were prepared with freshly doubly distilled water. The ionic strength (I) was adjusted to 0.1 M by addition of $NaClO_4$ (Sigma-Aldrich). Ethylene glycol was bought from Chempur (pure p.a.).

Mass spectrometric measurements

ESI-MS experiments were performed on the LCMS-9030 qTOF Shimadzu (Shimadzu, Kyoto, Japan) device, equipped with a standard ESI source and the Nexera X2 system. Analysis was performed in the positive ion mode between 100-1000 m/z. LCMS-9030 parameters: nebulizing gas - nitrogen, nebulizing gas flow 3.0 L/min, drying gas flow – 10L/min, heating gas flow – 10L/min, interface temperature 300 °C, desolvation line temperature – 400 °C, detector voltage – 2.02 kV, interface voltage 4.0 kV, collision gas - argon, mobile phase A) H2O +0.1% HCOOH, B) MeCN + 0.1% HCOOH, mobile phase total flow - 0.3 ml/min. The injection volume was optimized depending on the

intensity of the signals observed on the mass spectrum within the range of 0.1 to 1 μl. All obtained signals had a mass accuracy error in the range of 1 ppm. The concentration of peptide was 0.1 mM and M:L molar ratio was 0.9:1. Samples were prepared in the mixture of water/methanol (50/50 v/v) at pH 6.50. All of the used solvents were of LC-MS grade. The obtained data were analyzed by LabSolutions software (Shimadzu, Kyoto, Japan).

Potentiometric measurements

Potentiometric measurements were performed at a constant temperature of 25 °C under an argon atmosphere using a Molspin pH meter equipped with a Mettler-Toledo InLab semi combined electrode and a micrometer syringe with a volume of 0.5 cm^3. Before each measurement, the electrode was calibrated by titration of $HClO_4$ having a concentration of 4 mM with 0.1 M NaOH. Stability constants for proton, Cu(II) and Zn(II) complexes were calculated from potentiometric titration curves registered over the pH range 2.5−11. The pH-metric titrations were performed in an aqueous solution of 4 mM $HClO_4$ at 0.1 M $NaClO_4$ as ionic strength. The titrant was a carbonate-free standard solution of NaOH. The ligand concentration was about 1 mM, and the metal-to-ligand ratio was 0.9:1. The exact concentrations and the purities of the ligand solutions were determined by the Gran method.[1] The standard potential and the slope of the electrode couple were computed by means of GLEE program.[2] The HYPERQUAD 2006 program was used for the stability constant calculations.[3] The speciation and competition diagrams were computed with the HYSS program.[4]

Spectroscopic studies

The absorption spectra were recorded on a Varian Cary 300 Bio spectrophotometer and circular dichroism (CD) measurements were obtained on a Jasco J-1500 CD spectropolarimeter. For both methods, spectra were collected over the 200–800 nm range using quartz cuvettes with 1 cm optical path length. The samples were prepared in a water solution containing 4 mM $HClO_4$ and 0.1 M $NaClO_4$. The concentrations of solutions used for UV−vis and CD spectroscopic studies were similar to those used in the potentiometric experiments. Additionally, the far-UV CD spectra in the wavelength of 180-250 nm with 0.01 cm optical path length were obtained for ligand and also for Cu(II) and Zn(II) complexes at metal-to-peptide molar ratio 0.9:1 in a water solution of 4 mM $HClO_4$ and 0.1 M $NaClO_4$, where the concentration of peptide was 0.4 mM.

Electron paramagnetic resonance (EPR) spectra were recorded for Cu(II) complexes at 77 K at an X-band frequency (9.5 GHz) on a Bruker ELEXSYS E500 CW-EPR spectrometer equipped with an ER 036TM NMR Teslameter and E41 FC frequency counter. The peptide was prepared in an water solution containing 4 mM $HClO_4$ and 0.1 M $NaClO_4$, with 30% ethylene glycol as a cryoprotectant. The concentration of Cu(II) was 1 mM with a 0.9:1 metal-to-peptide molar ratio. The EPR parameters were determined from computer simulations of the experimental spectra using Bruker's WIN-EPR SIMFONIA Software, Version 1.2 (Billerica, USA).[5] Spectra were recorded in the range of pH 3-11. The pH was adjusted with appropriate amounts of $HClO_4$ and NaOH solutions.

DFT calculations

Computational methods of theoretical chemistry have been used as useful tool to predict structure and stability of the ligands and complexes.[6,7,8,9,10,11,12] Molecular orbital studies on Cu(II) and Zn(II) cations 1:1 complex with pHpG-4 peptide have been done on the DFT level of theory with IEFPCM[13] solvent (water) model introduced upon potential energy surface investigation. The integral equation formalism for polarizable continuum model (IEFPCM) approximation describes a solvent as a homogeneous dielectric medium with electrical permeability (ε) equal to that of a pure solvent, and the cavity size is modeled for a solvent immersed molecule. The starting structure of the peptide for DFT calculations was generated on the basis of the amino acid sequence after 75 ps simulation at 300 K, without cutoffs using BIO+ implementation of CHARMM force field. DFT calculations were performed with Gaussian 09 C.01[14] suite of programs using the ωB97X-D[15] long-range corrected hybrid density functional with damped atom-atom dispersion corrections was used with double-zeta 6-31G(d,p) basis set.

RESULTS AND DISCUSSION

Electrospray ionization mass spectrometry (ESI-MS) was used to determine the stoichiometry of Cu(II) and Zn(II) complexes revealing only equimolar complex species both in case of Cu(II)-pHpG-4 and Zn(II)-pHpG-4 (Figure S1, Figure S2). The most abundant peaks with m/z at 546.62, 682.77 and 910.03 in the Cu(II)- pHpG-4 spectrum have been assigned to the $[CuL]^{5+}$, $[CuL]^{4+}$ and $[CuL]^{3+}$ species (each with one potassium adduct), respectively. In case of ZnL system, signals from the complexes are much less intense than those observed in the case of the copper complexes. Peaks observed at 546.42, 682.52 and 910.03 correspond to $[ZnL+K]^{5+}$, $[ZnL+K]^{4+}$ and $[ZnL+K]^{3+}$, respectively. Experimental and simulated spectra for chosen signals (Figures S1 and S2, graphs A and B) were in good agreement. Other peaks come from differently charged species and also from their sodium and potassium adducts.

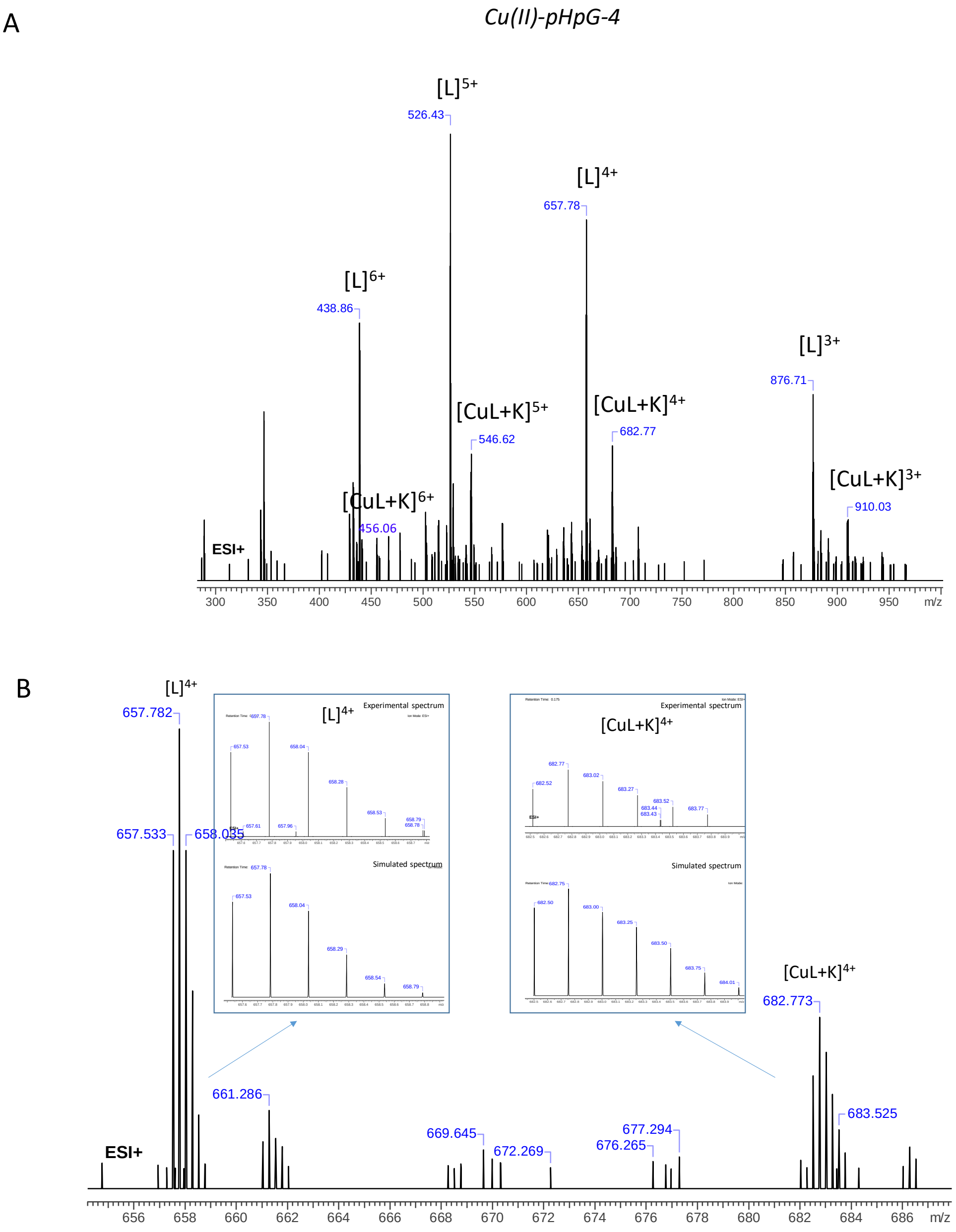

Figure S1. *ESI positive mass spectra (ESI-MS) for Cu(II)-VDHpHpG complex under the whole measuring range m/z (A) and for chosen signals (with experimental and simulated spectra) for [L]$^{4+}$ and [CuL+K]$^{4+}$(B). Metal:ligand in a 0.9:1 stoichiometry, where [ligand]$_{tot}$= 0.1 mM. Measurements were prepared in 1:1 MeOH-H_2O mixture at pH 6.50.*

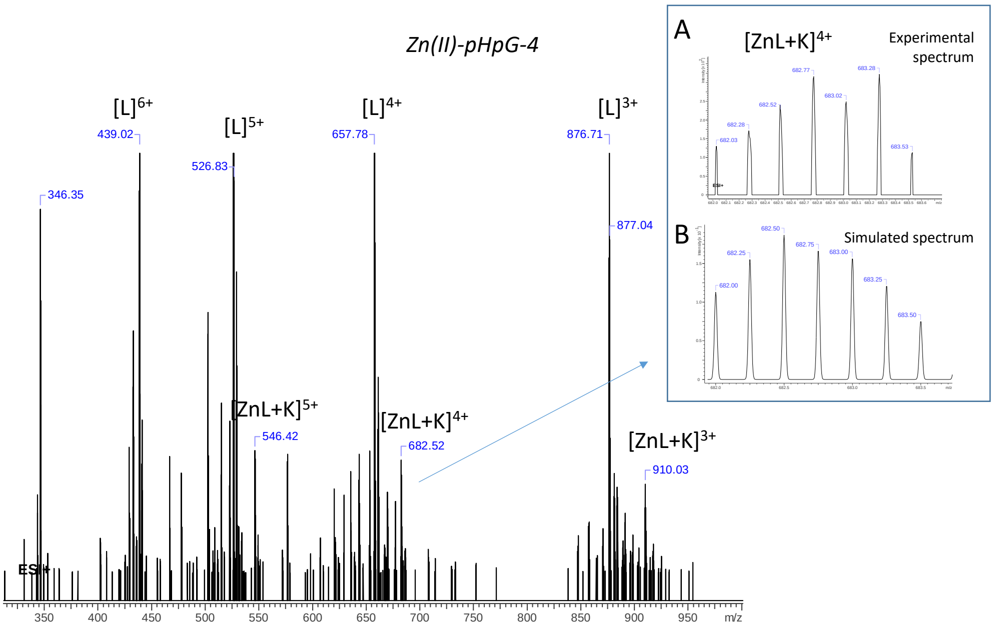

Figure S2. *ESI positive mass spectra (ESI-MS) for Zn(II)-pHpG-4 complex with an experimental (A) and simulated (B) spectra for chosen signal: $[ZnL+K]^{4+}$.Metal:ligand in a 0.9:1 stoichiometry, where $[ligand]_{tot}$= 0.1 mM. Measurements were prepared in 1:1 MeOH-H_2O mixture at pH 6.50.*

The acid-base properties of pHpG-4 and its coordination abilities towards copper(II) and zinc(II) ions were determined using a series of potentiometric titrations and spectroscopic methods (UV-vis, CD and EPR - for Cu(II) complexes). Information on structural properties were enriched with far-UV CD spectra and DFT calculations.

On the basis of a series of potentiometric titrations, twelve protonation constants were determined for the pHpG-4 ligand (VDHDHDHHHHHHPGSSVGGGGGGGGGGA-NH_2) (Figure S3). Three of them are derived from the carboxyl groups of Asp residues (p*Ka* range: 3.06 - 4.37), eight with the p*Ka* from 5.47 to 7.99 assigned to the imidazole rings of His residues, and a p*Ka* value of 9.18 to the N-terminal amino group. The ligand is completely deprotonated at pH 10 (Figure S3). The obtained values of the protonation constants are consistent with the generally recognized and common literature values. The wide range of p*Ka* values assigned to His residues is also known, especially for peptides rich in histidyl residues.[16,17,18,19,20]

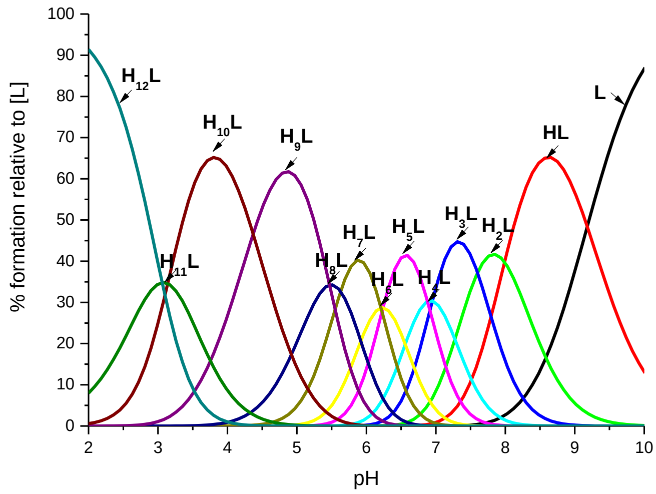

Figure S3. *Representative distribution diagram for the protonation of pHpG-4 peptide. Measurement were performed at a constant temperature of 25°C under an argon atmosphere using a Molspin pH-meter, equipped with a semi-combined electrode Mettler Toledo InLab and micrometer syringe with a volume of 0.5 cm^3. The pH-metric titrations were performed in a water solution of $HClO_4$ at 0.1 M $NaClO_4$. The titrant was a carbonate-free, standard solution of NaOH. $C°_L$ = 1mM*

Cu(II)- VDHDHDHHHHHHPGSSVGGGGGGGGGA-NH_2 (pHpG-4) complexes

Potentiometric titrations under given experimental conditions (molar ratio M:L – 0.9:1, millimolar concentrations) showed ten equimolar Cu(II)-pHpG-4 complexes characterized by the log*β* and p*Ka* values collected in Table S1. The distribution diagram for the obtained Cu(II)−pHpG-4 complex species is shown in Figure S4.

Table S1. *Potentiometric and spectroscopic data for proton and Cu(II)-pHpG-4 complexes in a water solution of 4 mM $HClO_4$ at I = 0.1 M ($NaClO_4$) and at 25°C (potentiometric titrations, UV-vis and CD spectroscopy) and at 77 K in water/ethylene glycol solution (EPR spectroscopy); [L] = 1 mM; Cu(II) to ligand ratio of 0.9:1.*

Potentiometry					UV-vis			CD		EPR		Proposed donors
species	log*β*	p*Ka*	pH		λ [nm]	ε [$M^{-1}cm^{-1}$]		λ [nm]	Δε [$M^{-1}cm^{-1}$]	A|| [G] (A_{zz})	g|| (g_z)	
HL	9.18(1)	9.18										
H_2L	17.17(1)	7.99										
H_3L	24.77(2)	7.60										
H_4L	31.72(3)	6.95										
H_5L	38.60(3)	6.88										
H_6L	44.84(3)	6.24										
H_7L	51.05(3)	6.21										
H_8L	56.68(3)	5.63										
H_9L	62.15(3)	5.47										

$H_{10}L$	66.52(3)	4.37										
$H_{11}L$	69.66(5)	3.14										
$H_{12}L$	72.72(6)	3.06										
CuH_7L	58.48(3)		4.10		624	66.50				161.80	2.31	$2N_{im}$
CuH_6L	54.06(1)	4.42	4.50		608	84.10		293<br>320<br>566	0.05<br>0.17<br>0.01	178.00	2.27	$2N_{im}$
CuH_5L	49.34(3)	4.72	5.15		577	87.50		278<br>310<br>498<br>584	-0.19<br>0.27<br>0.33<br>0.01			$2N_{im}$,<br>$1N_{am}$
CuH_4L	43.73(3)	5.61	5.70		561	97.23		312<br>498<br>605	0.33<br>0.52<br>0.09	186.10	2.25	$1N_{im}$,<br>$2N_{am}$
CuH_3L	37.78(3)	5.95	6.20		548	101.50		267<br>312<br>498<br>583	-1.94<br>0.61<br>0.58<br>0.05			$1N_{im}$,<br>$2N_{am}$
CuH_2L	31.23(4)	6.55	6.60		535	114.55				186	2.249	$1N_{im}$,<br>$2N_{am}$
CuHL	24.58(3)	6.65	6.90					271<br>312<br>477<br>554	-2.60<br>1.29<br>0.57<br>-0.21			$1N_{im}$,<br>$2N_{am}$,<br>NH_2<br>(ATCUN)
CuL	17.37(5)	7.21	7.30		524	130.10		272<br>310<br>475<br>555	-2.82<br>1.76<br>0.54<br>-0.48	200	2.185	$1N_{im}$,<br>$2N_{am}$,<br>NH_2<br>(ATCUN)
$CuH_{-1}L$	10.03(3)	7.34	7.80		523	141.90		271<br>310<br>477<br>552	-2.88<br>1.95<br>0.63<br>-0.68			$1N_{im}$,<br>$2N_{am}$,<br>NH_2<br>(ATCUN)
$CuH_{-2}L$	1.93(5)	8.10	9.90		523	145.00		272<br>310<br>476<br>553	-2.89<br>1.95<br>0.63<br>-0.68			$1N_{im}$,<br>$2N_{am}$,<br>NH_2<br>(ATCUN)

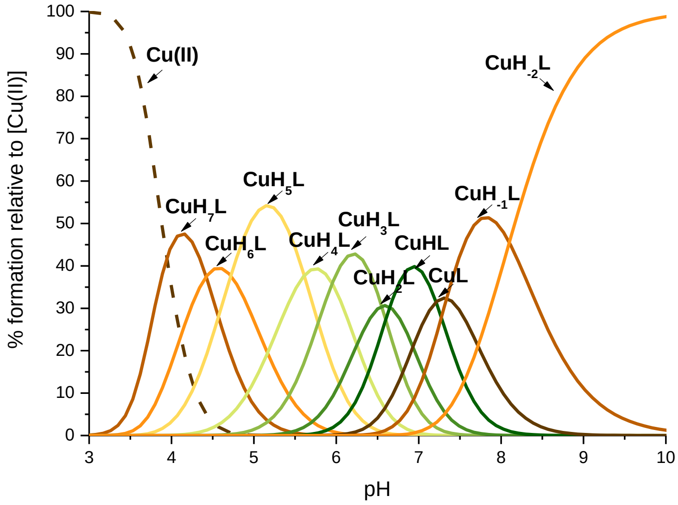

Figure S4. *Representative distribution diagram for the complex-formation of Cu(II)-pHpG-4, at 25°C and I = 0.1 M (NaClO$_4$). [L] = 1 mM; M:L molar ratio 0.9:1.*

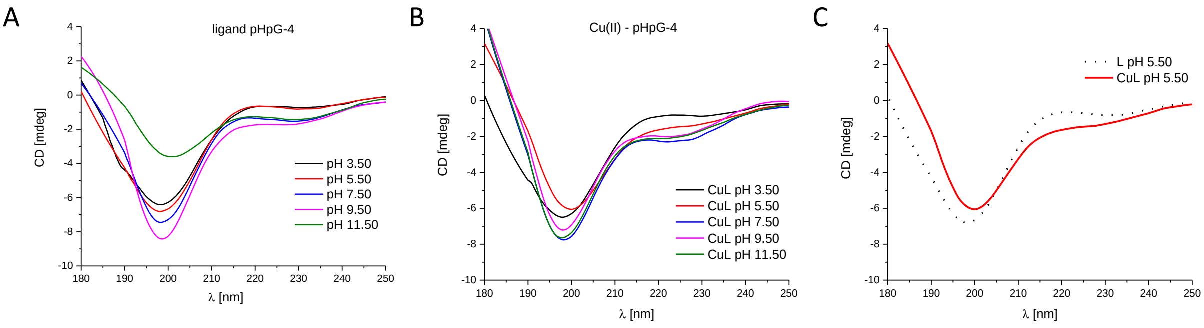

Figure S5. *The far-UV CD spectra for the pHpG-4 ligand (A) and Cu(II)-pHpG-4 system (B) at different pH vaules in a water solution of 4 mM HClO$_4$ at I = 0.1M (NaClO$_4$) and at 25°C; [L] = 0.4 mM; Cu(II) to ligand ratio of 0.9:1; additionally the comparison of spectra at pH 5.50 (C).*

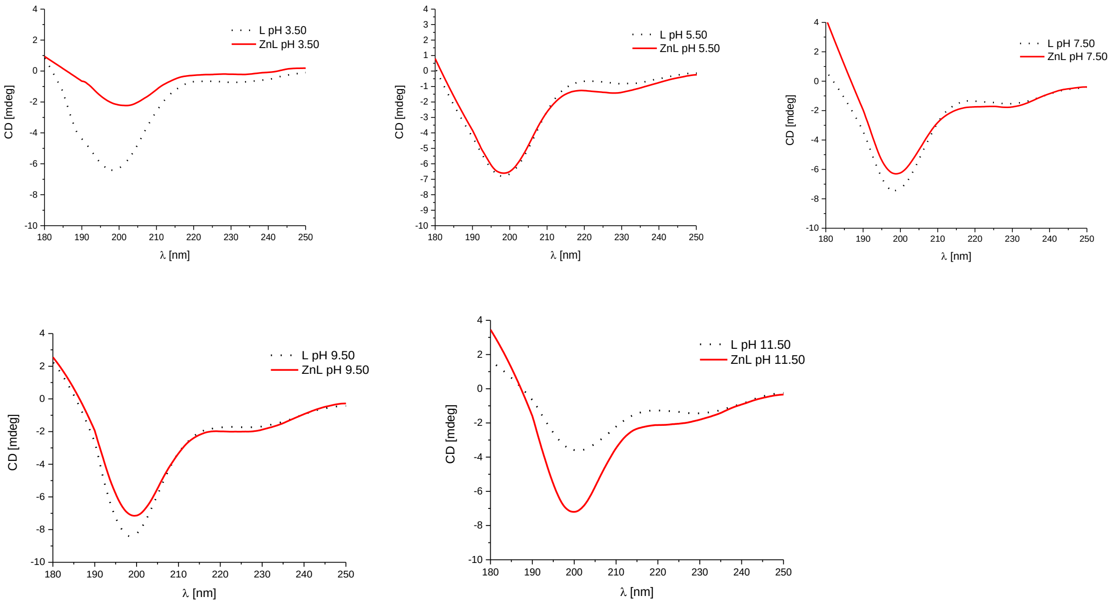

Figure S6. *The far-UV CD spectra for the pHpG-4 ligand (dropped lines) and Zn(II)-pHpG-4 system (red lines) at different pH vaules in a water solution of 4 mM $HClO_4$ at I = 0.1M ($NaClO_4$) and at 25°C; [L] = 0.4 mM; Zn(II) to ligand ratio of 0.9:1.*

CuH_7L is the first complex detected at acidic pH with maximum concentration at pH 4. In this complex, most probably two imidazole residues are involved in the copper(II) binding. The {$2N_{im}$} coordination mode is supported by (i) EPR parameters ($A_{||}$ = 161.80 and $g_{||}$ = 2.31) (Table S1, Figure S7 and Table S2)[21] and (ii) the d-d band at 624 nm in the UV-vis spectrum (Figure S8A). Lack of pronounced changes in spectroscopic parameters for the next species, CuH_6L, dominating at pH 4.5, suggests the same type of coordination {$2N_{im}$}. On the other hand, a significant difference in p*Ka* values for the complex and for the corresponding ligand species (p*Ka* for CuH_6L = 4.4 and p*Ka* for H_7L = 6.21, respectively), while the spectroscopic parameters remain unchanged, may suggests the presence of two complex species in equilibrium, where two different His residues bind Cu(II) ion. A similar phenomenon was observed in our previous works on the specificity of metal complexes with peptides such as His-tag6 or pHG, containing the poly-His motif in the sequence.[16,17]

With the increase of pH and the occurrence of the next complex species, CuH_5L, the maximum of absorption in the spectrum was shifted towards shorter wavelengths in the range of d-d transitions (608 nm → 577 nm, Figure S8A) and the new signal with a positive Cotton effect ($\Delta\varepsilon$ = 0.33 at 498 nm) in the CD spectrum occurred (Figure S8B). These changes suggest the binding of the copper(II) ion by an additional nitrogen atom, possibly from an amide bond (negative band at 278 nm).[22] The {3N} coordination mode is much clearer at about pH 5.7, where the next complex species, CuH_4L dominates in solution. Changes in the EPR parameters: i) the increase of the value of $A_{||}$ parameter: 178.00 →

186.10 and ii) the decrease of the value of the $g_{||}$ parameter: 2.27 → 2.25 strongly confirm the coordination of the copper ion by the three nitrogen donors (Table S1, Figure S6 and Table S2). Additionally, a slight shift of the maximum absorption to shorter wavelengths with the intensity increasing at pH 5.7 in the UV-vis spectrum and also an increasing of the band intensity at 312 nm in the CD spectrum may suggest a change in the coordination mode from {$2N_{im}$, $1N_{am}$} to {$1N_{im}$, 2 N_{am}}.

For the next complex species: CuH_3L and CuH_2L, no significant changes in spectroscopic parameters were observed. The p*Ka* values of these complex species are close to those of the corresponding ligand species, which suggests the non-binding deprotonation of imidazole residues (p*Ka* CuH_3L = 5.95 (6.24), p*Ka* for CuH_2L = 6.55 (6.88)) and thus no changes in the coordination mode.

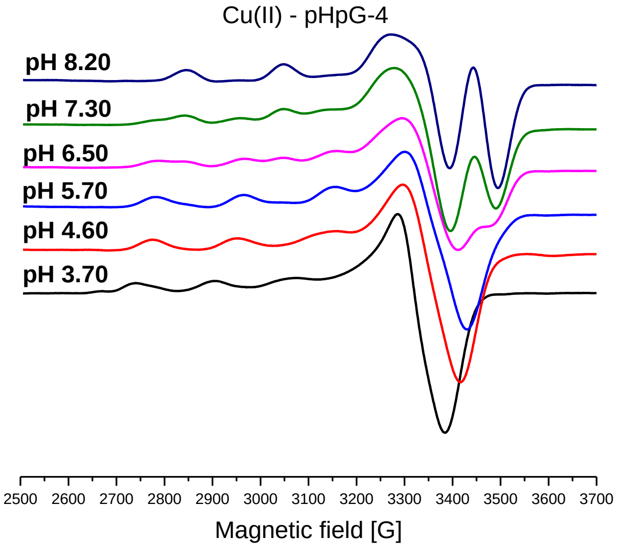

Figure S7. *X-band EPR spectra of frozen solution (77 K) at different pH values, for the system Cu(II)-pHpG-4. pHpG-4 peptide in water solution of 4mM $HClO_4$ at I=0.1 M ($NaClO_4$). [Cu(II)] = 1mM; M:L molar ratio 0.9:1. Ethylene glycol (30%) was used as a cryoprotectant. Nuclear spin of copper (^{63}Cu and ^{65}Cu nuclei): 3/2; nuclear spin of nitrogen: 1. The ^{63}Cu and ^{65}Cu nuclei give rise to a characteristic pattern because of electron spin–nuclear spin hyperfine interactions.*

Table S2. *EPR parameters with number of proposed nitrogen donors. The given parameters were simulated using WinEPR SimFonia Version 1.2 (Billerica,USA). Nuclear spin of copper: 3/2; nuclear spin of nitrogen: 1; MW Frequency 9.6195-9.6260 GHz*

pH	$A_{||}$ [G] (A_{zz})	$g_{||}$ (g_z)	$g_{\perp}$ (g_x=g_y)	MW Frequency	Proposed donors
3.9	161	2.31	2.06	9.6260	2N
4.5	178	2.27	2.05	9.6231	2N
5.7	186	2.25	2.045	9.6233	3N
6.5	186	2.249	2.045	9.6245	3N
7.3	200	2.185	2.04	9.6195	4N
8.2	202	2.185	2.04	9.6260	4N

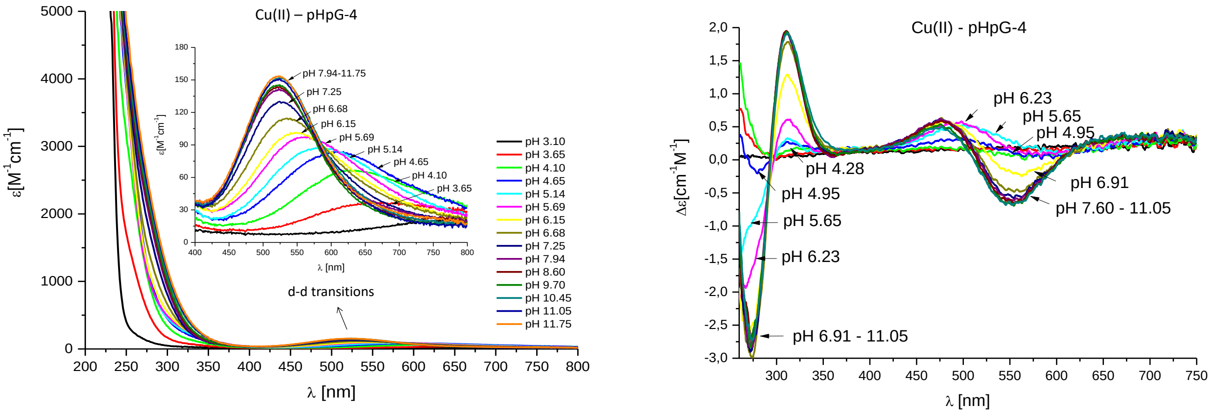

Figure S8. *UV-vis absorption (A) and circular dichroism (B) spectra at different pH values for the Cu(II)-pHpG-4 system in water solution of 4 mM $HClO_4$ at I = 0.1 M ($NaClO_4$) and 25°C. [L] = 1 mM; M:L molar ratio 0.9:1. Path length 1 cm.*

At a pH around 7, in which the CuHL complex species dominate in solution, a signal with the negative Cotton effect at 554 nm appears in the CD spectrum (Figure S8B). The simultaneous presence of signals with the positive and negative Cotton effects in the range of d-d transition bands suggests the formation of a {4N} complex with a square-planar geometry. The CD spectrum also shows a signal at 272 nm suggesting nitrogen coordination from the N-terminus of the peptide, completing the copper(II) coordination sphere. At this pH, a complex with a {$1N_{im}$, $2N_{am}$, $1NH_2$} donor set is formed, what is characteristic for systems with Cu(II) (or Ni(II)), and peptides with a free N-terminus and histidine in the third position. This coordination mode dominates above pH 6.9 (including CuHL, CuL, $CuH_{-1}L$ and $CuH_{-2}L$ complex species) and is also supported by: i) EPR parameters values: $A_{||}$ = 200 and $g_{||}$ = 2.185 and ii) maximum absorbance at around 523 nm in the UV-vis spectra (Table S2, Figure S7, FigureS 8A). No significant changes in the far-UV CD spectra were observed with increasing pH value (Figure S5B) indicating no structural changes.

Zn(II) - VDHDHDHHHHHHPGSSVGGGGGGGGGGA-NH_2 (pHpG-4) complexes

In the case of complexes with Zn(II) ions, seven differently protonated complex species (ZnH_6L, ZnH_5L, ZnH_4L, ZnH_3L, ZnH_2L, ZnHL, ZnL) were determined in the range of pH 5-10 (Table S3, Figure S9). Based on a series of potentiometric titrations, the mode of zinc(II) coordination by the studied peptide was proposed. Initially, Zn(II) is bound to two His residues (in the ZnH_6L complex species), and, with the increase of pH, subsequent imidazole residues become coordinated (ZnH_5L - $3N_{im}$; ZnH_4L - $4N_{im}$). It is not excluded, that the {$4N_{im}$} coordinated species may be in equilibrium with other complexes, in which two different His residues coordinate the Zn(II) ion. Further complexes come from the deprotonation of unbound imidazole residues and the N-terminal amino group.

Table S3. *Potentiometric data for proton and Zn(II)-pHpG-4 complexes in a water solution of 4 mM $HClO_4$ at I = 0.1M ($NaClO_4$) and at 25°C; [L] = 1 mM; Zn(II) to ligand ratio of 0.9:1.*

Species	logβ	log*K*	Proposed donors
ZnH_6L	49.51(5)		$2N_{im}$
ZnH_5L	44.11(3)	5.40	$3N_{im}$
ZnH_4L	38.30(3)	5.81	$4N_{im}$
ZnH_3L	31.59(3)	6.71	$4N_{im}$
ZnH_2L	24.21(4)	7.38	$4N_{im}$
ZnHL	15.82(5)	8.39	$4N_{im}$
ZnL	6.56(6)	9.26	$4N_{im}$

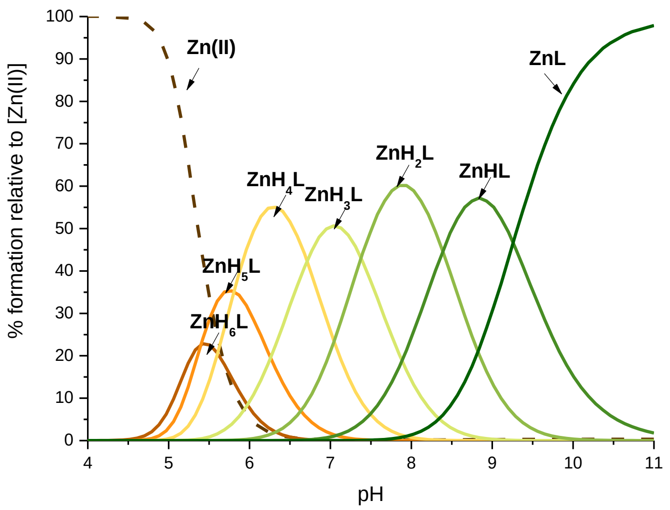

Figure S9. *Representative distribution diagram for the complex-formation of Zn(II)-pHpG-4 in a water solution of 4 mM $HClO_4$ at I = 0.1M ($NaClO_4$) and at 25°C; [L] = 1 mM; Zn(II) to ligand ratio of 0.9:1.*

DFT calculations complete the experimental results

The Cu(II) - pHpG-4 complexes

Theoretical studies perfectly complement experimental work and often allow to explain the phenomenon of metal ion - peptide coordination. Studies of some of the Cu(II) complexes, compared with the Cu(II) - pHpG-4 system, were also supplemented by DFT calculations and allowed to accurately determine the interactions between metal-peptide.[16,17,23]

In case of Cu(II)-pHpG-4 system, seven Cu(II) complexes have been found. CuH_7L and CuH_6L are 2N type and use H3 and H5 imidazole rings to bind the metal cation. The bond lengths are similar: 1.829 +/- 0.03 Å (Table S4). Interestingly, short, 6 residue (19-24) unusual polyglycine helical fragments can be detected in CuH_6L and CuH_7L complexes (marked in red in Figure S10), similar to ones reported before.[24]

The first 3N type complex is CuH_5L; its binding pattern is similar to the previously mentioned complexes: two imidazole nitrogens from H3 and H5 bind Cu(II) with bond lengths 1.851 Å and 1.856 Å; the third nitrogen comes from the amide of H3, which is 2.101 Å away from the central Cu(II) ion. CuH_4L, CuH_3L and CuH_2L are 3N type complexes that share the same 3N binding pattern, in which D2 and H3 amide nitrogens and the H3 imidazole nitrogen are involved. The Cu(II) – amide nitrogen bond lengths are in the range 1.85-1.89 Å, and the Cu(II) - imidazole bond is moderately longer - 2.06 Å. CuHL is a typical albumine-like complex. The Cu(II) binding to amides of D2 and H3 (1.874 Å and 1.873 Å bond lengths, respectively) and to H3 imidazole nitrogen (2.167 Å bond length) is supported by the binding of the V1 amino nitrogen (bond length 2.241 Å).

All metal – ligand distances are shown in the Table S4.

Table S4. *Cu(II) – ligand distances in angstroms.*

X	CuH_7L (2N)	CuH_6L (2N)	CuH_5L (3N)	CuH_4L (3N)	CuH_3L (3N)	CuH_2L (3N)	CuHL (4N)
Cu..X							
N (amino V1)							2.241
N (amide D2)				1.891	1.869	1.849	1.874
N (H3)	1.831	1.826	1.851	2.173	2.134	2.067	2.167
N (amide H3)			2.101	1.862	1.848	1.846	1.873
N (H5)	1.830	1.827	1.856				

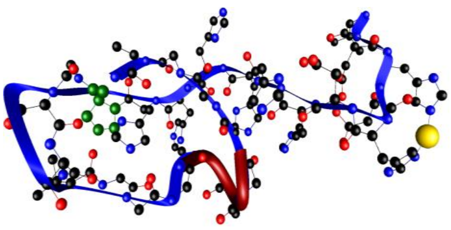

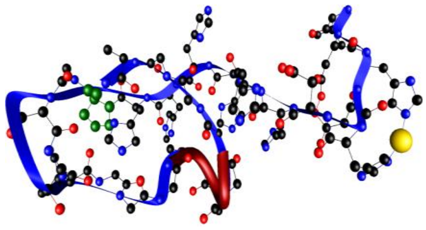

CuH$_7$L
N (imidazole His3), N (imidazole His5)

CuH$_6$L
N (imidazole His3), N (imidazole His5)

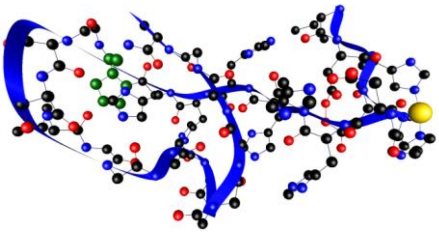

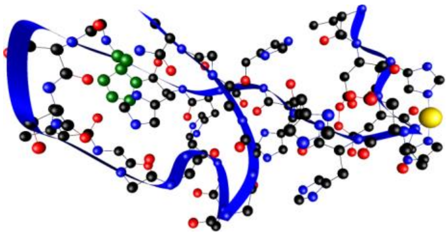

CuH$_5$L
N (imidazole His3), N (amide His3), N (imidazole His5)

CuH$_4$L
N (amide D2), N (imidazole His3), N (amide His3)

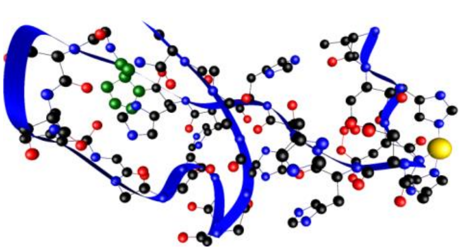

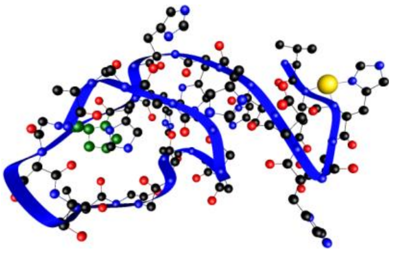

CuH$_3$L
N (amide D2), N (imidazole His3), N (amide His3)

CuH$_2$L
N (amide D2), N (imidazole His3), N (amide His3)

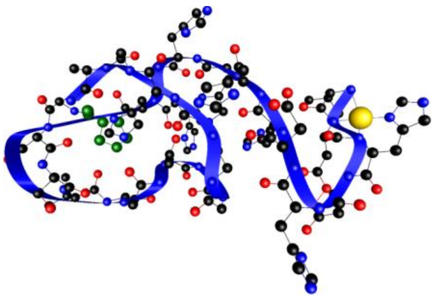

CuHL
N (amine V1), N (amide D2), N (imidazole His3), N (amide His3)

Figure S10. *Structures of Cu(II)-VDHDHDHHHHHHPGSSVGGGGGGGGGGA-NH$_2$ complexes. Blue ribbons follow backbone, the proline is marked in green.*

The Zn(II) - (pHpG-4) complexes

Four Zn(II) complexes have been found (Figure S11).

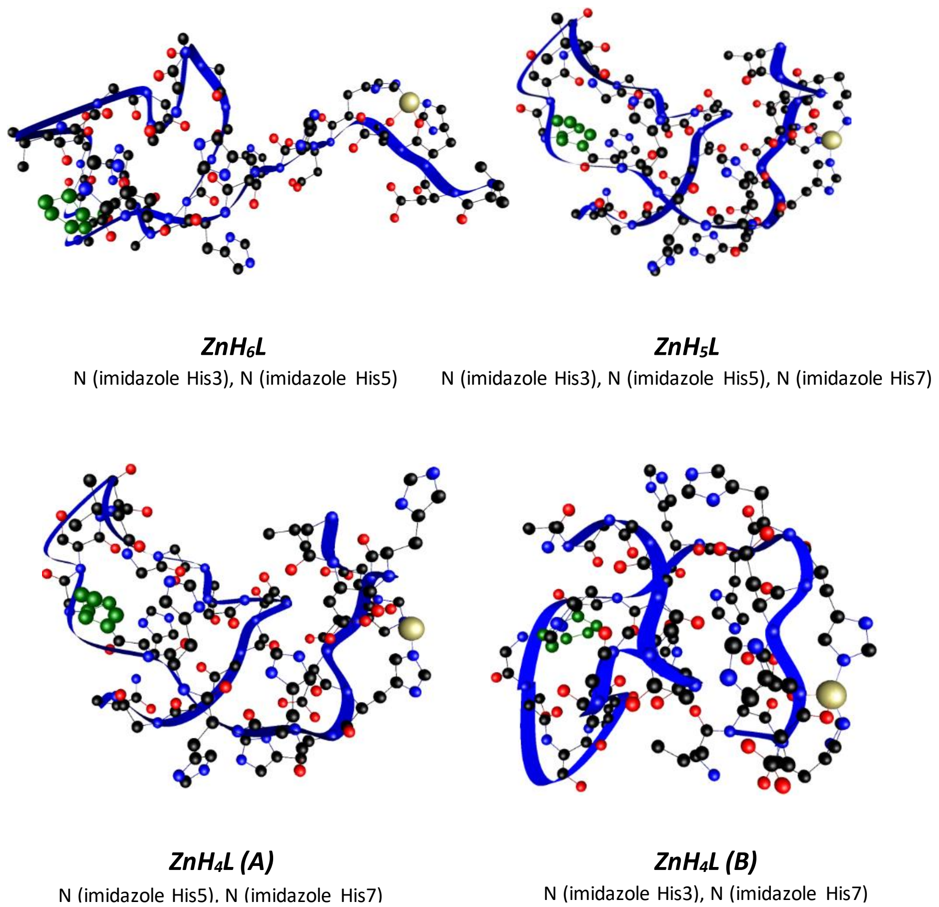

Figure S11. *Structures of Zn(II)-VDHDHDHHHHHHPGSSVGGGGGGGGGGA-NH_2 complexes. Blue ribbons follow backbone, the proline is marked in green.*

The ZnH_6L (2N) complex is based on two Zn(II) – imidazole interactions, where H3 and H5 take part in binding (with Cu(II)-N bond lengths of 2.016 Å and 1.955 Å, respectively. The ZnH_6L complex is additionally stabilized by two supporting interactions with carbonyl oxygens of H3 and D4.

The ZnH_5L complex is the only one in which three imidazole rings (of H3, H5 and H7) are involved in Zn(II) binding, with bond lengths of 1.989 Å, 1.963 Å and 1.989 Å, respectively. The N-type set of interactions is supported by one Zn-O bond (2.090 Å), where the oxygen comes from the carbonyl group of H3.

Interestingly, the ZnH_4L species can exist in two forms (A and B), where imidazole rings of the H5 (1.930 Å) and H7 (1.912 Å) for ZnH_4L (A) and H3 (1.999 Å) and H7 (1.950 Å) for ZnH_4L (B) are involved. Both forms display one Zn-O supporting interaction with bond length ~1.96 Å. All metal-ligand interactions are presented in the Table S5.

Table S5. *Zn – ligand distances in angstroms.*

X	ZnH_6L (2N)	ZnH_5L (3N)	ZnH_4L (A) (2N)	ZnH_4L (B) (2N)
N (H3)	2.016	1.989		1.999
N (H5)	1.955	1.963	1.930	
N (H7)		1.989	1.912	1.950
O (C=O H3)	2.107	2.090		
O (C=O D4)	2.043			2.019
O (C=O D6)			1.961	
O (COO D2)				1.964

All Zn(II) complexes locate the metal at the D2-H7 region of the ligand. It is worth to note that in ZnH_6L, there is a semi-linear fragment in the metal binding area, and surprisingly, proline is not involved in such build as one could expect (does not form the specific kink). Due to the presence of the semi-linear fragment in ZnH_6L, the metal is relatively more exposed to further interactions in comparison to the rest of the investigated complexes.

<u>Thermodynamic stability of snake venom derived complexes</u>

<u>Cu(II) complexes</u>

Competition plot for Cu(II) complexes, which is a hypothetical simulation (based on the calculated stability constants) of a system in which equimolar amounts of metal ions and ligands are present and pH-dependent apparent affinity (pK_D) values (at pH 7.4 and also at pH 5.4 - typical for snake venom glands)[25] may give us additional information about interaction specificity with the pHpG-4 peptide and other peptides containing the free N-terminus (Figure S12A, Table S6), showed that below pH 7, pHpG-$1_{Ath.sq}$ (EDDHHHHHHHHHGVGGGGGGGGG-NH_2) is most effective in copper(II) binding, while the least effective peptide is pHpG-4 (VDHDHDHHHHHHPGSSVGGGGGGGGGGA-NH_2). pHpG-$1_{Ath.sq}$ has a higher affinity towards Cu(II) than the pHpG-4 peptide containing the ATCUN motif, and most likely, this is due to the possibility of creating polymorphic states by pHpG-1 peptide and forming a helical structure in the presence of metal ions.[18] The plot shows that above pH 7, the situation changes and Cu(II) ions bind more effectively to the pHpG-4 peptide, which can be explained by metal coordination through the albumin-like binding, which begins to dominate at around pH 7 (CuHL species). The shorter analog of pHpG-4, without Val in the first position and poly-Gly domain in the sequence, N-DpH (DHDHDHHHHHHPGSSV-NH_2), turns out to be the most effective in Cu(II) binding below pH 4.5 and the least effective above pH 7.5.

A comparison of the Cu(II) ion binding efficiency by pHpG-4 and C- and N-protected peptides containing poly-His and/or poly-Gly motifs (Figure S12B) clearly show that about pH 8, almost 100% of the copper ions are bound to the peptide containing the ATCUN motif. At acidic pH values, the

copper ion binding efficiency of the peptides decreases with the decreasing number of available His residues in the peptide sequences, which is in good agreement with our chemical expectations.

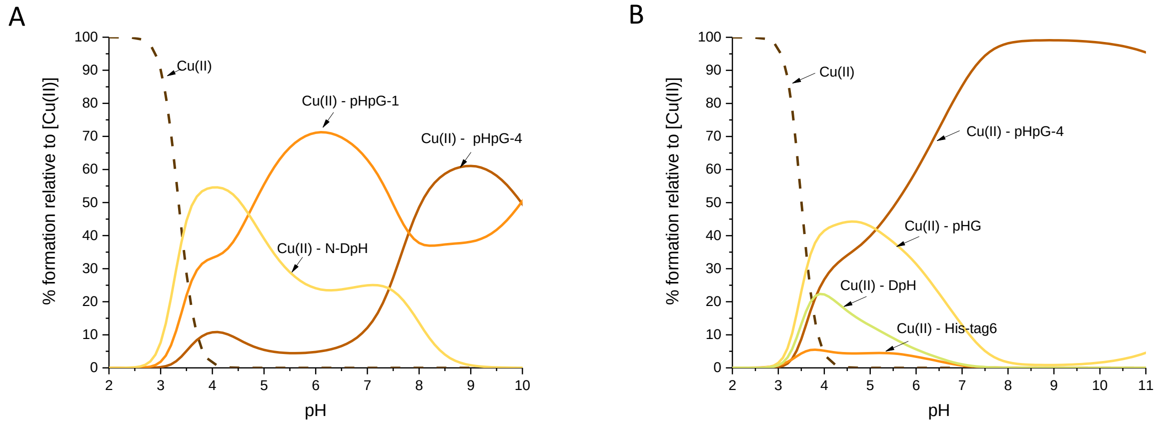

Figure S12. *Competition plot (hypothetical simulation (based on the calculated stability constants) of a system in which equimolar amounts of metal ions and ligands are present) with peptides containing poly-His motifs. (A) complexes with N-terminal free peptides (EDDHHHHHHHHHGVGGGGGGGGG-NH_2, pHpG-1; DHDHDHHHHHHPGSSVGGGGGGGGGGA-NH_2, pHpG-4; DHDHDHHHHHHPGSSV-NH_2, N-DpH; (B) complexes with N- and C-terminally protected peptides (VDHDHDHHHHHHPGSSVGGGGGGGGGGA-NH_2, pHpG-4; Ac-EDDHHHHHHHHHG-NH_2, pHG; Ac-DHDHDHHHHHHPGSSV-NH_2, DpH; Ac-HHHHHH-NH_2, His-tag6. The potentiometric data of compared peptides are taken from* [16,17,23,18]

Table S6. *pH-dependent apparent affinity values for the Cu(II)-peptide systems compared in the competition plots.*

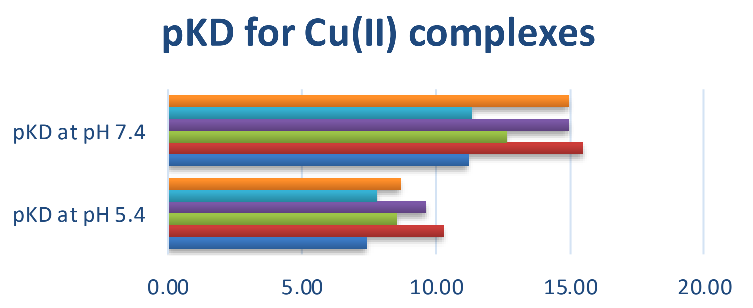

	pKD at pH 5.4	pKD at pH 7.4
pHpG-4	8.69	14.96
DpH	7.80	11.35
N-DpH	9.64	14.97
pHG	8.56	12.65
pHpG-1	10.29	15.51
His-tag6	7.42	11.22

Comparison of copper ion complexes with the studied peptide from *Echis ocellatus* venom and other peptides containing the ATCUN motif found in the literature showed that the pHpG-4 peptide forms the most thermodynamically stable complexes with Cu(II) ions in the whole pH range (Figure S13). The competition plot shows, *i.a.* that DAHQ is more efficient in Cu(II) binding than the DAH peptide, but less efficient than pHpG-4, mainly due to the number of histidine residues in the peptide sequences - the more His residues in the sequence, the stronger the binding, even despite the similar coordination

pattern, as in the case of DAHQ and pHpG-4 peptides - up to two imidazole residues bind the Cu(II) ion at pH 4.5. However, it is worth to note that the aforementioned peptides start to form typical albumin-like binding at different pH values: in the case of DAH – around pH 4, in Cu(II)-DAHQ – at pH 5, and in the case of the Cu(II)-pHpG-4 complex – almost 3 units higher (at pH around 6.9) than in the first system.[26,27] Most likely, polymorphic binding sites present in the Cu(II)-pHpG-4 complex (and similar poly-His peptides) efficiently prevent amide binding at lower pH.

To conclude, the presence of i) proline, ii) poly-Gly, iii) ATCUN motif and number of His residues in the pHpG-4 sequence strongly stabilizes the Cu(II) complexes (above pH 7).

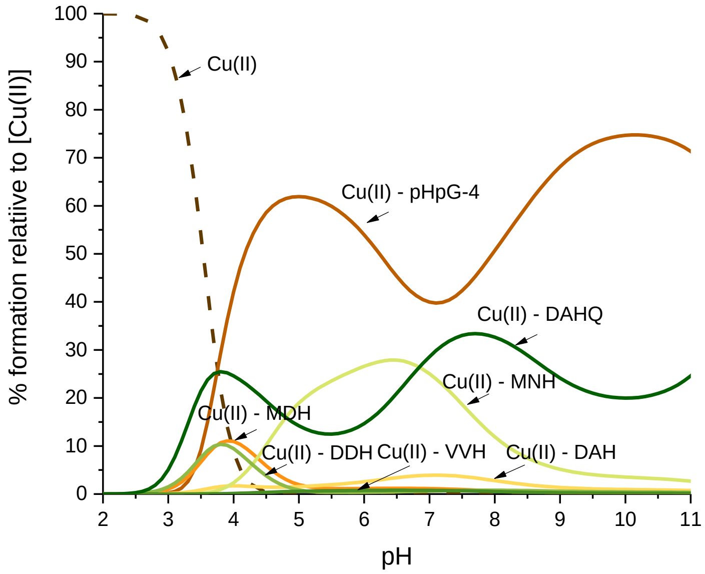

Figure S13. *Competition plot (hypothetical simulation (based on the calculated stability constants) of a system in which equimolar amounts of metal ions and ligands are present) with peptides containing ATCUN motif: tripeptides: MNH-NH$_2$ (MNH), MDH-NH$_2$ (MDH), DAH-NH$_2$ (DAH), peptides containing poly-Gly motif: VVHGGGGG-NH$_2$ (VVH), DDHGGGGG-NH$_2$ (DDH) and with unblocked octapeptide containing ATCUN motif and additionally three histidines arranged in the HXHXHX motif – DAHQERMDVSETHLHWHT (DAHQ). The potentiometric data of compared peptides are collected in Table S7.*

Table S7. *Protonation and stability constants of peptides containing ATCUN motif and their Cu(II) complexes compared with Cu(II)- pHpG-4 system. Constants are presented as cumulative logβ_{jk} ones.[a] Standard deviations of the last digits are given in parentheses, at the values obtained directly from the experiment. L stands for a peptide with acid-base active groups. Values in italics correspond to pK_a values of the peptides and were derived from cumulative constants.[b]*

Species	logβ_{jk}[a]					
	MNH-NH$_2$ (MNH)[28]	MDH-NH$_2$ (MDH)[26]	DAH-NH$_2$ (DAH)[26]	VVHGGGGG-NH$_2$ (VVH)[29]	DDHGGGGG-NH$_2$ (DDH)[29]	DAHQERMDVSETHLHWHT (DAHQ)[27]
HL	7.305(4)<br>*7.31*	7.625(9)<br>*7.63*	7.68(1)<br>*7.68*	7.77(1)<br>*7.77*	8.18(1)<br>*8.18*	8.12(0)<br>*8.12*
H$_2$L	13.526(4)	13.77(1)	14.18(1)	13.89(1)	14.83(1)	15.49(0)

	6.22	*6.24*	*6.50*	*6.12*	*6.65*	*7.36*
H_3L	-	16.23(2)<br>*2.46*	17.11(1)<br>*2.93*	-	18.52(1)<br>*3.68*	22.39(1)<br>*6.90*
H_4L	-	-	-	-	20.79(3)<br>*2.28*	28.81(0)<br>*6.42*
H_5L	-	-	-	-	-	34.65(0)<br>*5.84*
H_6L	-	-	-	-	-	39.22(1)<br>*4.56*
H_7L	-	-	-	-	-	43.20(1)<br>*3.98*
H_8L	-	-	-	-	-	46.56(0)<br>*3.36*
H_9L	-	-	-	-	-	49.45(1)<br>*2.89*
$H_{10}L$	-	-	-	-	-	51.61(1)<br>*2.16*
CuH_6L	-	-	-	-	-	42.69(3)
CuH_4L	-	-	-	-	-	34.90(2)
CuH_3L		-	-	-	-	30.26(2)
CuH_2L			-	-	-	25.74(1)
CuHL	-	-	-	-	19.85(1)	20.62(1)
CuL	-	8.93(8)	8.48(1)	-	9.70(1)	14.69(2)
$CuH_{-1}L$	-	-	-	2.78(2)	4.60(1)	8.15(3)
$CuH_{-2}L$	-0.03(1)	-1.26(1)	-0.61(1)	-1.32(1)	-0.93(1)	0.77(3)
$CuH_{-3}L$	-	-	-	-	-	-10.29(4)

[a] $\beta(H_jL_k) = [H_jL_k]/([H]^j[L]^k)$, in which [L] is the concentration of the fully deprotonated peptide.

[b] $\log\beta(H_jL_k) - \log\beta(H_{j-1}L_k) = pK_a$

Zn(II) complexes

In the competition plot for Zn(II) complexes, the situation looks different when compared to Cu(II) systems. The Zn(II)-pHpG-4 complex forms the least stable complexes in comparison to other peptides (Figure S14A, Table S8). Thus, it can be suggested that the presence of the Pro residue and the poly-Gly sequence in the peptide can significantly reduce the thermodynamic stability of zinc(II) complexes. The competition plot in the Figure S14B shows an opposite trend in the affinity of metal ion coordination to the one observed in case of Cu(II) complexes with the same peptides which is probably related to the availability of metal binding sites. In case of pHpG-4, the $4N_{im}$ binding mode could be 'blocked' by the flexible poly-Gly sequence. In the N-DpH peptide, the poly-His motif is more accessible for Zn(II) ions

(Figure S14B, Table S8). Despite the availability of oxygen donors from the three aspartic acid residues in case of N-DpH peptide, which are also capable to bind zinc[30], the more preferred Zn(II) binding sites are the imidazole nitrogen and the nitrogen from N-terminal amine.

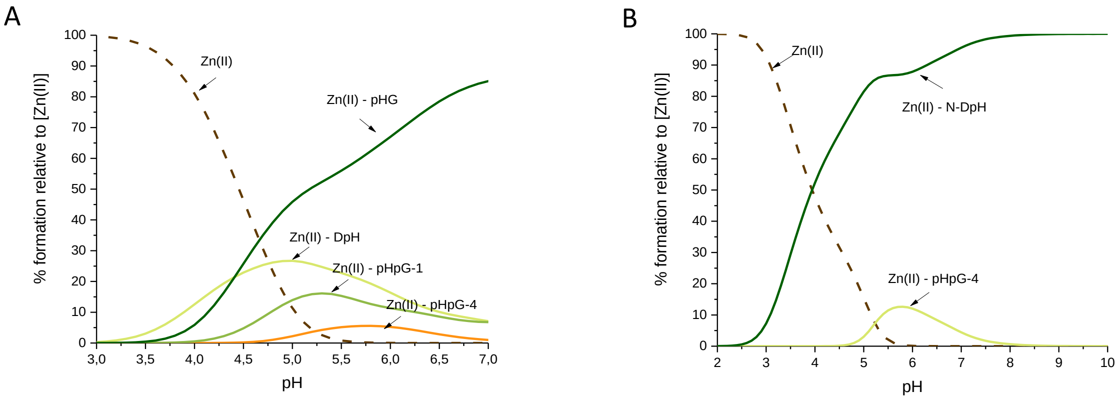

Figure S14. *Competition plot (hypothetical simulation (based on the calculated stability constants) of a system in which equimolar amounts of metal ions and ligands are present) with peptides containing poly-His motifs. Complexes with A) N-terminal free peptides: VDHDHDHHHHHHPGSSVGGGGGGGGGGA-NH_2 (pHpG-4), EDDHHHHHHHHHGVGGGGGGGGGG-NH_2 (pHpG-1) and C- and N- terminally protected peptides: Ac-DHDHDHHHHHHPGSSV-NH_2 (DpH), Ac-EDDHHHHHHHHHG-NH_2 (pHG) and B) N-terminal free peptides: VDHDHDHHHHHHPGSSVGGGGGGGGGGA-NH_2 (pHpG-4) and DHDHDHHHHHHPGSSV-NH_2 (N-DpH). The potentiometric data of compared peptides are taken from* [16,23,18,31]

Table S8. *pH-dependent apparent affinity values for the Zn(II)-peptide systems compared in the competition plots.*

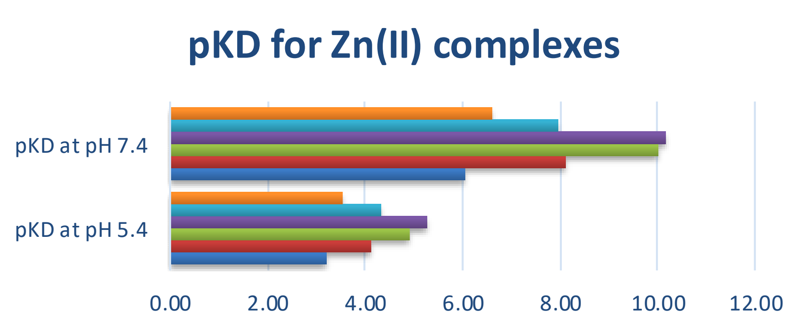

	pKD at pH 5.4	pKD at pH 7.4
pHpG-4	3.54	6.61
DpH	4.33	7.96
N-DpH	5.27	10.17
pHG	4.91	10.02
pHpG-1	4.12	8.12
His-tag6	3.21	6.05

REFERENCES

(1) Gran, G. DETERMINATION OF THE EQUIVALENT POINT IN POTENTIOMETRIC TITRATIONS. *Acta Chemica Scandinavica* **1950**, *4* (4), 559-577, Article. DOI: 10.3891/acta.chem.scand.04-0559.

(2) Gans, P.; O'Sullivan, B. GLEE, a new computer program for glass electrode calibration. *Talanta* **2000**, *51* (1), 33-37, Article. DOI: 10.1016/s0039-9140(99)00245-3.
(3) Gans, P.; Sabatini, A.; Vacca, A. SUPERQUAD - AN IMPROVED GENERAL PROGRAM FOR COMPUTATION OF FORMATION-CONSTANTS FROM POTENTIOMETRIC DATA. *Journal of the Chemical Society-Dalton Transactions* **1985**, (6), 1195-1200. DOI: 10.1039/dt9850001195.
(4) Alderighi, L.; Gans, P.; Ienco, A.; Peters, D.; Sabatini, A.; Vacca, A. Hyperquad simulation and speciation (HySS): a utility program for the investigation of equilibria involving soluble and partially soluble species. *Coordination Chemistry Reviews* **1999**, *184*, 311-318. DOI: 10.1016/s0010-8545(98)00260-4.
(5) Weber, R. T., WIN-EPR SimFonia, Software version 1.2, EPR Division (Bruker; Instruments, I., Billerica, USA, 1995).
(6) Mielke, Z.; Latajka, Z.; Olbert-Majkut, A.; Wieczorek, R. Matrix infrared spectra and ab initio calculations of the nitrous acid complexes with nitrogen monoxide. *Journal of Physical Chemistry A* **2000**, *104* (16), 3764-3769, Article. DOI: 10.1021/jp993889i.
(7) Wieczorek, R.; Latajka, Z.; Lundell, J. Quantum chemical study of the bimolecular complex of HONO. *Journal of Physical Chemistry A* **1999**, *103* (31), 6234-6239, Article. DOI: 10.1021/jp990610p.
(8) Olszewski, T. K.; Wojaczynska, E.; Wieczorek, R.; Bakowicz, J. alpha-Hydroxyphosphonic acid derivatives of 2-azanorbornane: synthesis, DFT calculations, and crystal structure analysis. *Tetrahedron-Asymmetry* **2015**, *26* (12-13), 601-607, Article. DOI: 10.1016/j.tetasy.2015.04.012.
(9) Salvador, P.; Wieczorek, R.; Dannenberg, J. J. Direct calculation of trans-hydrogen-bond C-13-N-15 3-bond J-couplings in entire polyalanine alpha-helices. A density functional theory study. *Journal of Physical Chemistry B* **2007**, *111* (9), 2398-2403, Article. DOI: 10.1021/jp064706c.
(10) Rudowska, M.; Wieczorek, R.; Kluczyk, A.; Stefanowicz, P.; Szewczuk, Z. Gas-Phase Fragmentation of Oligoproline Peptide Ions Lacking Easily Mobilizable Protons. *Journal of the American Society for Mass Spectrometry* **2013**, *24* (6), 846-856, Article. DOI: 10.1007/s13361-013-0585-1.
(11) Gumienna-Kontecka, E.; Berthon, G.; Fritsky, I. O.; Wieczorek, R.; Latajka, Z.; Kozlowski, H. 2-(Hydroxyimino)propanohydroxamic acid, a new effective ligand for aluminium. *Journal of the Chemical Society-Dalton Transactions* **2000**, (22), 4201-4208, Article. DOI: 10.1039/b004432f.
(12) Stokowa-Soltys, K.; Barbosa, N. A.; Kasprowicz, A.; Wieczorek, R.; Gaggelli, N.; Gaggelli, E.; Valensin, G.; Wrzesinski, J.; Ciesiolka, J.; Kulinski, T.; et al. Studies of viomycin, an anti-tuberculosis antibiotic: copper(II) coordination, DNA degradation and the impact on delta ribozyme cleavage activity. *Dalton Transactions* **2016**, *45* (20), 8645-8658, Article. DOI: 10.1039/c6dt00245e.
(13) Cammi, R. Molecular Response Functions for the Polarizable Continuum Model: Physical Basis and Quantum Mechanical Formalism. *Molecular Response Functions for the Polarizable Continuum Model: Physical Basis and Quantum Mechanical Formalism* **2013**, 1-58, Book. DOI: 10.1007/978-3-319-00987-2.
(14) Frisch, M. J. e. a. Gaussian 09, Revision. *Gaussian, Inc., Wallingford CT, 2009*.
(15) Chai, J. D.; Head-Gordon, M. Long-range corrected hybrid density functionals with damped atom-atom dispersion corrections. *Physical Chemistry Chemical Physics* **2008**, *10* (44), 6615-6620, Article. DOI: 10.1039/b810189b.
(16) Watly, J.; Simonovsky, E.; Barbosa, N.; Spodzieja, M.; Wieczorek, R.; Rodziewicz-Motowidlo, S.; Miller, Y.; Kozlowski, H. African Viper Poly-His Tag Peptide Fragment Efficiently Binds Metal Ions and Is Folded into an alpha-Helical Structure. *Inorganic Chemistry* **2015**, *54* (16), 7692-7702, Article. DOI: 10.1021/acs.inorgchem.5b01029.
(17) Watly, J.; Simonoysky, E.; Wieczorek, R.; Barbosa, N.; Miller, Y.; Kozlowski, H. Insight into the Coordination and the Binding Sites of Cu2+ by the Histidyl-6-Tag using Experimental and Computational Tools. *Inorganic Chemistry* **2014**, *53* (13), 6675-6683, Article. DOI: 10.1021/ic500387u.
(18) Pontecchiani, F.; Simonovsky, E.; Wieczorek, R.; Barbosa, N.; Rowinska-Zyrek, M.; Potocki, S.; Remelli, M.; Miller, Y.; Kozlowski, H. The unusual binding mechanism of Cu(II) ions to the poly-histidyl domain of a peptide found in the venom of an African viper. *Dalton Transactions* **2014**, *43* (44), 16680-16689, Article. DOI: 10.1039/c4dt02257b.
(19) Hecel, A.; Watly, J.; Rowinska-Zyrek, M.; Swiatek-Kozlowska, J.; Kozlowski, H. Histidine tracts in human transcription factors: insight into metal ion coordination ability. *Journal of Biological Inorganic Chemistry* **2018**, *23* (1), 81-90, Article. DOI: 10.1007/s00775-017-1512-x.
(20) Tanaka, N.; Kawachi, M.; Fujiwara, T.; Maeshima, M. Zinc-binding and structural properties of the histidine-rich loop of Arabidopsis thaliana vacuolar membrane zinc transporter MTP1. *Febs Open Bio* **2013**, *3*, 218-224, Article. DOI: 10.1016/j.fob.2013.04.004.
(21) Peisach, J.; Blumberg, W. E. STRUCTURAL IMPLICATIONS DERIVED FROM ANALYSIS OF ELECTRON-PARAMAGNETIC RESONANCE-SPECTRA OF NATURAL AND ARTIFICIAL COPPER PROTEINS. *Archives of Biochemistry and Biophysics* **1974**, *165* (2), 691-708, Article. DOI: 10.1016/0003-9861(74)90298-7.

(22) Dzien, E.; Dudek, D.; Witkowska, D.; Rowinska-Zyrek, M. Thermodynamic surprises of Cu(II)-amylin analogue complexes in membrane mimicking solutions. *Scientific Reports* **2022**, *12* (1), 9, Article. DOI: 10.1038/s41598-021-04197-5.
(23) Watly, J.; Hecel, A.; Wieczorek, R.; Swiatek-Korlowska, J.; Kozlowski, H.; Rowinska-Zyrek, M. Uncapping the N-terminus of a ubiquitous His-tag peptide enhances its Cu2+ binding affinity. *Dalton Transactions* **2019**, *48* (36), 13567-13579, Article. DOI: 10.1039/c9dt01635j.
(24) Wieczorek, R.; Dannenberg, J. J. Hydrogen-bond cooperativity, vibrational coupling, and dependence of helix stability on changes in amino acid sequence in small 3(10)-helical peptides. A density functional theory study. *Journal of the American Chemical Society* **2003**, *125* (46), 14065-14071, Article. DOI: 10.1021/ja034034t.
(25) Mackessy, S. P.; Baxter, L. M. Bioweapons synthesis and storage: The venom gland of front-fanged snakes. *Zoologischer Anzeiger* **2006**, *245* (3-4), 147-159, Article. DOI: 10.1016/j.jcz.2006.01.003.
(26) Mlynarz, P.; Valensin, D.; Kociolek, K.; Zabrocki, J.; Olejnik, J.; Kozlowski, H. Impact of the peptide sequence on the coordination abilities of albumin-like tripeptides towards Cu2+, Ni2+ and Zn2+ ions. Potential albumin-like peptide chelators. *New Journal of Chemistry* **2002**, *26* (2), 264-268, Article. DOI: 10.1039/b107412c.
(27) Janicka-Klos, A.; Juszczyk, P.; Grzonka, Z.; Kozlowski, H. Competition between the albumin and three histidine fragment of amyloid precursor protein sites to bind Cu(II). *Polyhedron* **2008**, *27* (5), 1511-1516, Article. DOI: 10.1016/j.poly.2008.01.025.
(28) Bossak, K.; Drew, S. C.; Stefaniak, E.; Plonka, D.; Bonna, A.; Bal, W. The Cu(II) affinity of the N-terminus of human copper transporter CTR1: Comparison of human and mouse sequences. *Journal of Inorganic Biochemistry* **2018**, *182*, 230-237, Article. DOI: 10.1016/j.jinorgbio.2018.01.011.
(29) Miyamoto, T.; Kamino, S.; Odani, A.; Hiromura, M.; Enomoto, S. Basicity of N-Terminal Amine in ATCUN Peptide Regulates Stability Constant of Albumin-like Cu2+ Complex. *Chemistry Letters* **2013**, *42* (9), 1099-1101, Article. DOI: 10.1246/cl.130405.
(30) Ben-Shushan, S.; Miller, Y. Molecular Mechanisms and Aspects on the Role of Neuropeptide Y as a Zn2+ and Cu2+ Chelator. *Inorganic Chemistry* **2021**, *60* (1), 484-493, Article. DOI: 10.1021/acs.inorgchem.0c03350.
(31) Remelli, M.; Brasili, D.; Guerrini, R.; Pontecchiani, F.; Potocki, S.; Rowinska-Zyrek, M.; Watly, J.; Kozlowski, H. Zn(II) and Ni(II) complexes with poly-histidyl peptides derived from a snake venom. *Inorganica Chimica Acta* **2018**, *472*, 149-156, Article. DOI: 10.1016/j.ica.2017.05.070.
